# Construction of novel cGMP FRET-sensors based on PKG from Plasmodium falciparum

**DOI:** 10.1186/2050-6511-16-S1-A34

**Published:** 2015-09-02

**Authors:** Gaia Calamera, Andrea Hembre Ulsund, Ornella Manfra, Jeong Joo Kim, Choel Kim, Finn Olav Levy, Kjetil Wessel Andressen

**Affiliations:** 1 Department of Pharmacology, University of Oslo and Oslo University Hospital, Oslo, Norway; 2 K.G. Jebsen Cardiac Research Centre and Center for Heart Failure Research, University of Oslo, Oslo, Norway; 3 Department of Pharmacology, Baylor College of Medicine, Houston, TX, USA

## Background

Several sensors for cyclic nucleotides have been developed the past decade. However, there are few sensors for cGMP available and even fewer that detect low concentrations of cGMP. Currently, only the CFP/YFP sensors Cygnet 2.1, cGES-DE5 and cGi-500 (EC50 ~1.7 µM, 1.5 µM and 500 nM, respectively) and the T-sapphire/Dimer2 sensor Red cGES-DE5 (EC50 ~40 nM) are available [1-3]. We aim to measure localized pools of cGMP in single adult cardiac myocytes, and have previously found that such sensors should have an EC50 less than 50 nM, thus excluding the CFP/YFP sensors above. The Red cGES-DE5 has an acceptable EC50, but tagging this sensor to locate in distinct compartments within cardiac myocytes yielded sensors with a smaller dynamic range. We therefore decided to construct new FRET-based cGMP-sensors with high affinity for cGMP that could be better candidates for subcellular localization.

## Methods

Based on the recent crystallization of Plasmodium falciparum cGMP-dependent Protein Kinase [4], sensors for cGMP were constructed using the cyclic nucleotide-binding domain D flanked with different FRET-pairs (either CFP/Citrine or T-sapphire/Dimer2) in its N- and C-terminal to generate two different sensors for cGMP, excitable at various wavelengths. Sensors were then expressed in HEK293 cells and their activity tested in vitro by measuring FRET upon addition of cGMP.

## Results

Sensors with high affinity for cGMP (EC50 of 26 ± 14 nM) were constructed. We also determined that the affinity for cAMP was almost 50 fold lower (1 ± 0.5 µM).

## Conclusion

We have constructed FRET-based cGMP sensors with high affinity for cGMP that can be utilized in single cells when concentrations of cAMP are less than 0.1 µM.
